# Bias assessment and correction for Levin’s population attributable fraction in the presence of confounding

**DOI:** 10.1007/s10654-023-01063-8

**Published:** 2024-01-03

**Authors:** John Ferguson, Alberto Alvarez, Martin Mulligan, Conor Judge, Martin O’Donnell

**Affiliations:** HRB Clinical Research Facility Galway, University of Galway, Galway, Ireland

**Keywords:** Population attributable fraction, Levins formula, Miettinens formula

## Abstract

In 1953, Morton Levin introduced a simple approach to estimating population attributable fractions (PAF) depending only on risk factor prevalence and relative risk. This formula and its extensions are still in widespread use today, particularly to estimate PAF in populations where individual data is unavailable. Unfortunately, Levin’s approach is known to be asymptotically biased for the PAF when the risk factor-disease relationship is confounded even if relative risks that are correctly adjusted for confounding are used in the estimator. Here we describe a simple re-expression of Miettinen’s estimand that depends on the causal relative risk, the unadjusted relative risk and the population risk factor prevalence. While this re-expression is not new, it has been underappreciated in the literature, and the associated estimator may be useful in estimating PAF in populations when individual data is unavailable provided estimated adjusted and unadjusted relative risks can be transported to the population of interest. Using the re-expressed estimand, we develop novel analytic formulae for the relative and absolute asymptotic bias in Levin’s formula, solidifying earlier work by Darrow and Steenland that used simulations to investigate this bias. We extend all results to settings with non-binary valued risk factors and continuous exposures and discuss the utility of these results in estimating PAF in practice.

**Supplementary Information:**

The online version contains supplementary material available at 10.1007/s10654-023-01063-8.

## Introduction

The Population Attributable Fraction (PAF), sometimes also referred to as the Population Attributable Risk and the Excess Fraction, is a commonly used metric in epidemiology. It is useful both to ascertain the importance of a risk factor in causing disease as well as to identify the best risk factors to target in a health intervention.

Applications of attributable fractions abound in the literature starting with Richard Doll’s work in estimating the burden of lung cancer due to smoking [1], with recent examples relating to modifiable risk factors of gout in the US [2], risk factors for depression in Brazil [3] and modifiable risk factors for cancer in Denmark [4], with these studies representing only a very small sampling of recent literature.

Intuitively, PAF represents the fraction of prevalent disease cases that might have been avoided in some population if a risk factor were not present.^1^ If we express disease prevalence in the current population as $P(Y=1)$, $Y$ being a binary indicator for disease, and the prevalence of disease that would have been observed in the target population had the risk factor been removed as $P({Y}_{0}=1)$, PAF can be expressed as:

1 $$PAF=\frac{P(Y=1)-P({Y}_{0}=1)}{P(Y=1)}.$$

Here, we are restricting our usage of the term risk factor to apply to environmental, physiological or behavioural determinants of a particular disease; that is we do not view variables that have non-causal associations with disease as risk factors. Such factors might be binary, multi-level or continuously distributed. As an example, depending on the resolution of data capture, smoking might be coded as a binary indicator for current smoking, separate indicators for whether an individual is currently smoking, has given up smoking or has never smoked, or perhaps as total nicotine intake via smoking. Definition (1) and its interpretation, in terms of a comparison between the current population and the population had the risk factor been removed, when this is possible at least hypothetically, is valid for any type of exposure distribution. However, the details of the estimation process will differ between these three situations. We first will discuss the simplest case where the risk factor is binary before extending to more general situations.

In the binary risk factor setting, a vast literature has evolved on methods to estimate (1) with individual-level data [5]. However, it is sometimes necessary to derive estimates of PAF in scenarios where the collection of individual-level data is impossible. As an example, the Global Burden of Disease project [6] estimate population attributable fractions at a country level for differing risk factor/disease combinations. For certain countries, no individual-level data linking risk factors of interest to disease may be available. The most common approach in this situation is to substitute the estimated risk factor prevalence in the population, $\widehat{\uppi }$, and estimated causal relative risk of disease (adjusted for known confounders), $\widehat{R{R}_{C}}$,^2^ into Eq. (2) below. Equation (2) was introduced by Morton Levin [7], also in the context of estimating the PAF for smoking as a cause of lung cancer:

2 $$PA{F}_{L}=\frac{\pi (R{R}_{C}-1)}{1+\pi (R{R}_{C}-1)}.$$

(2) is prominent throughout the literature on PAF estimation. However, as recognised by multiple authors [8–11], (2) will usually differ from (1) as a quantity. In particular (2) will equal the true PAF only under the unlikely condition that the association between risk factor and outcome is unconfounded, or more technically exhibits marginal exchangeability [12]. Marginal exchangeability implies that the causal relative risk, $R{R}_{C}=\frac{P({Y}_{1}=1)}{P({Y}_{0}=1)}$, defined as the ratio of disease prevalence comparing scenarios where the entire population was exposed and the entire population was unexposed to the risk factor in question equals the unadjusted relative risk, $R{R}_{U}=\frac{P(Y=1|X=1)}{P(Y=1|X=0)}$. See Supplementary Material Section 1B for more discussion.

## Miettinen’s definition of PAF

While (2) does not equal PAF in the absence of this ‘no confounding’ assumption, an alternative expression introduced by Miettinen [13], which involves the prevalence of the exposure within cases, ${\pi }_{c}$, as opposed to the overall population prevalence does equal (1) irrespective of the degree of confounding. Miettinen’s expression is given by

3 $$PA{F}_{M}={\pi }_{c}\frac{R{R}_{C}-1}{R{R}_{C}},$$

where ${\pi }_{c}$ is now the proportion of disease cases that have the risk factor.

The above expression is correct (that is $PA{F}_{M}=PAF$) provided the causal relative risk, $R{R}_{C}$, is constant in differing joint strata of the confounder variables. Otherwise, a similar formula holds, replacing the causal relative risk in the overall population with the causal relative risk in individuals exposed to the risk factor (See the worked example later in this section for an example, and Supplementary Section S2 for a proof). The usual advice is to use (3) when possible [9] and not (2) as a basis for estimating PAF. However, estimates for $\pi$ are more likely to be found in the published literature than estimates for ${\pi }_{c}$ and as a result, researchers are more likely to use (2) in practice. For example, the Global Burden of Disease project utilise (2) rather than (3) to estimate PAF [6].

## A re-expression of Miettinen’s PAF

A fact that has been underappreciated in the literature is that Eq. (3) can be re-expressed to be a function of three quantities: the prevalence of the risk factor in the population, $\pi$, the causal relative risk, $R{R}_{C}$, and the unadjusted relative risk, $R{R}_{U}$ in the population:

4 $$PA{F}_{M}=\left[\frac{\pi R{R}_{U}}{1+\pi (R{R}_{U}-1)}\right]\frac{R{R}_{C}-1}{R{R}_{C}}$$

The equality of (3) and (4) can be shown by applying Bayes’ rule (supplementary section S3). This formula was known to Miettinen (see Eq. 8 in his 1974 paper), but has received little attention in the literature, although an equivalent formula was recently described by Susuki and Yamamoto [14]. While a somewhat simple re-expression of (3), (4) may be practically useful in estimating PAF with summary data as unadjusted relative risks are often reported together with adjusted relative risks. It is useful to notice that under no confounding, which implies $R{R}_{U}=R{R}_{C}$, the expression (4) simplifies to Levin’s formula. This observation implies that in addition to Levin’s formula being correct under marginal exchangeability as mentioned previously, it is also correct under a slightly weaker assumption of no-confounding in risk ratio ($R{R}_{U}=R{R}_{C}$), together with an assumption of no effect modification across confounder strata. However, formulae (2) and (4) will otherwise differ.

## A worked example demonstrating the bias in Levin's formula

For illustration regarding both the biases at play from Levin’s approach, we consider a hypothetical example with a single 2-level confounder, physical activity, that causally the risk factor affects a binary risk factor, hypertension, and disease. The physical activity/hypertension strata in the population, the population proportions in each stratum and the resulting observed probabilities of disease in the population are given in Table 1.

**Table 1:** Hypothetical population where a single binary confounder, physical activity, affects the both a risk factor, hypertension, and disease

Subgroup	Proportion of population within subgroup (%)	Confounder stratum (physical activity)	Risk factor level (hypertension)	Observed probability of disease	Counterfactual probability of disease under no hypertension
1	50	High	No	0.1	0.1
2	30	High	Yes	0.15	0.1
3	5	Low	No	0.2	0.2
4	15	Low	Yes	0.3	0.2

The table shows the proportions of individuals in each of the 4 physical activity/hypertension subgroups, the observed probabilities of disease in each of these subgroups, and the counterfactual probabilities of disease in each subgroup if hypertension were absent from the population

First, we will calculate the PAF from definition (1). The observed probability of disease in the population is just a weighted average of the observed probabilities of disease in the differing physical activity/hypertension subgroups, with weighting according to the corresponding proportions in the population: $P\left(\mathrm{Y}=1\right)=$ 0.5(0.1) + 0.3(0.15) + 0.05(0.2) + 0.15(0.3) = 0.15. To calculate the counterfactual probability of disease under elimination of hypertension, we need to work out the counterfactual probabilities of disease in each subgroup, under the intervention that hypertension is eliminated. In the subgroups of the population that had no hypertension (subgroups 1 and 3), this is the same as the observed probability of disease. In the subgroups of the population with hypertension and a particular level of physical activity, this is the probability of disease in the subgroup having the same level of physical activity and no hypertension (This calculation is making use of the conditional exchangeability or no unmeasured confounding assumption). The counterfactual probability of disease in the population, under elimination of hypertension pressure is then a similar weighted average of these subgroup counterfactual probabilities. That is: $P({Y}_{0}=1)=0.5(0.1)+0.3(0.1)+0.05(0.2)+0.15(0.2)=0.12$. The PAF can then be calculated from Eq. (1) as $\frac{P(Y=1)-P({Y}_{0}=1)}{P(Y=1)}=\frac{0.15-0.12}{0.15}=0.2$.

Since, physical activity is the only confounder of the risk factor/disease relationship, causal relative risks can be calculated by the taking the ratio of the probabilities of disease for individuals with high and low blood pressure, within the same stratum of physical activity. These probabilities are (0.15 vs 0.1) in the high-physical activity stratum, and (0.3 vs 0.2) in the low-physical activity stratum, both leading to the same stratum-specific causal relative risk for hypertension, $R{R}_{C}=1.5$.

The unadjusted relative risk in the population can be worked out by comparing the observed probabilities of disease in individuals with and without hypertension. Individuals with hypertension constitute 45% of the overall population, 2/3 of which (30% of the overall population) have high physical activity and 1/3 of which have low physical activity. This indicates that the observed probability of disease within individuals with hypertension is a (2/3, 1/3) weighted average: (2/3) 0.15 + (1/3) 0.3 = 0.2, of the observed probabilities of disease in the subgroup with hypertension and high physical activity and the observed probability of disease in the subgroup with hypertension and low physical activity. In a similar, way we can deduce that the observed probability of disease within individuals with no hypertension is $\left( {{1}0/{11}} \right)0.{1 } + \, \left( {{1}/{11}} \right)0.{2 }\sim \, 0.{11}$. The unadjusted relative risk for hypertension is simply the ratio, 0.20/0.11 = 1.83, of these two probabilities. Finally the prevalence of hypertension is 30% + 15% = 45% (or 0.45 as a proportion).

Numerically, Levin’s formula works out to be: $PA{F}_{L}=\frac{\pi \left(R{R}_{C}-1\right)}{1+\pi \left(R{R}_{C}-1\right)}=\frac{0.45\left(1.5-1\right)}{1+0.45\left(1.5-1\right)}=0.184$, an underestimate of the PAF = 0.2. As we might expect Miettinen’s formula (as given in formula (4)) $PA{F}_{M}=\left[\frac{0.45\left(1.83\right)}{1+0.45\left(1.83-1\right)}\right]\frac{1.5-1}{1.5}=0.6\left(\frac{2}{3}\right)=0.2$, leads to the correct PAF.

## Why does Levin’s approach fail?

One might ask, why has Levin’s approach failed here? As demonstrated in [15], Levin’s formula can be interpreted as a ratio of approximate ‘excess disease risk’, given by $\pi \left(R{R}_{C}-1\right)B$ among the proportion, $\pi ,$ of individuals exposed to the risk factor, compared to an assumed counterfactual baseline disease probability of B under the hypothetical scenario that nobody was exposed. Note that the total risk in the population (the denominator of Levin’s formula) can be approximated as baseline risk + approximate excess risk, which is $B+\pi \left(R{R}_{C}-1\right)B.$ The baseline risks, $B$, then cancel out on the numerator and denominator of Levin’s formula. However, this calculation only holds true if there is no confounding. In contrast, in the above example individuals who have hypertension are more likely to have low physical activity. Because low physical activity increases disease risk, these individuals will have a larger counterfactual risk, ${B}{\prime}>B$ under elimination of hypertension compared to the counterfactual risk in the general population, that is $B$, if hypertension were eliminated. The true excess risk in individuals with hypertension is actually $\pi \left(R{R}_{C}-1\right){B}{\prime}>\pi \left(R{R}_{C}-1\right){B},$ and as a result Levin’s formula, in this example, underestimates the PAF. In contrast, Miettinen’s formula is correct. Miettinen’s formula can be interpretated as saying the proportion of disease cases that would be prevented by elimination of the risk factor (that is the PAF) is the proportion of disease cases that are exposed to the risk factor, which is $0.6$, times the relative decrease in disease probability in those risk-factor exposed individuals had the risk factor been eliminated, which is: $\frac{R{R}_{C}-1}{R{R}_{C}}=\frac{2}{3}$. This alternative construction that deals with relative (and not baseline) disease probabilities, applied solely to the subgroup exposed to the risk to the risk factor, obviates problems with confounding.

## What if there is effect modification across strata?

In the above example, the casual relative risk is 1.5 in both confounder strata. If, in contrast, there is effect modification on the relative risk scale so that the causal risk factor varies among confounder strata, substituting the overall causal relative risk, $R{R}_{C}$ into (4), will no longer be correct. For instance, suppose the column ‘observed probability of disease’ in Table 1 was changed to have the entries (0.1, 0.15, 0.2, 0.6), with the population subgroup proportions unchanged. In this case, the per-stratum causal relative risks of hypertension among active and inactive individuals would be 1.5 and 3. The overall causal relative risk, comparing counterfactual scenarios where the entire population is exposed and is unexposed to the risk factor, is now $R{R}_{C}=2$. Substituting $R{R}_{C}=2$ into Eq. (4) leads to $PA{F}_{M}=0.346$, an underestimate of the correct PAF, which is 0.385 (Levin’s approach now generates $PA{F}_{M}=0.31$). Here the correct relative risk to use in (4) is the causal relative risk of hypertension, restricted to the sub-population exposed to hypertension, which can be worked out to be 2.25. Substituting this modified relative risk into Eq. (4) leads to the correct PAF. In practice, estimates for the causal relative risk in risk factor exposed individuals are usually unavailable from published summary data, and the best one can do is to estimate PAF by substituting estimated values for risk factor prevalence, $\pi$, unadjusted relative risk, $R{R}_{U}$, and the overall causal relative risk, $R{R}_{C}$, into (4). This will only be an asymptotically correct approach if there is no effect modification, but should behave better than Levin’s approach even if there is effect modification. In practice, $R{R}_{C}$ is usually estimated via a statistical modelling approach adjusting for known confounders.

## Analysis of bias in Levin’s estimate, assuming no effect modification on the relative risk scale

Darrow and Steenland [10] investigated the bias of Levin’s formula using simulation, under an assumption that there was no effect modification on the relative risk scale. They showed that as the degree of confounding, specified by ${\text{max}}\{C,1/C\}$ where $C=\frac{R{R}_{C}}{R{R}_{U}}$ gets larger, with no confounding in risk ratio being represented by $C=1$, the magnitude of *relative* bias in Levin’s formula will increase. Their simulations also indicated that this relative bias will be larger for smaller risk factor prevalences, as again observed in the previous example. However, having an explicit formula for relative bias allows a more rigorous analysis of limiting behaviour for extreme values of $C$ than was possible in [10].

Given the formula (4), which correctly identifies the $PAF$ when causal relative risks do not vary over confounder strata, an analytic expression for the relative asymptotic bias can be trivially derived by simply taking the ratio of (3) and (4). After some simplification (supplementary section S4) this results in:^3^

5 $$\frac{PA{F}_{L}}{PAF}=\frac{1+\pi (R{R}_{U}-1)}{1+\pi (C\times R{R}_{U}-1)}\times C$$

**Fig. 1:**
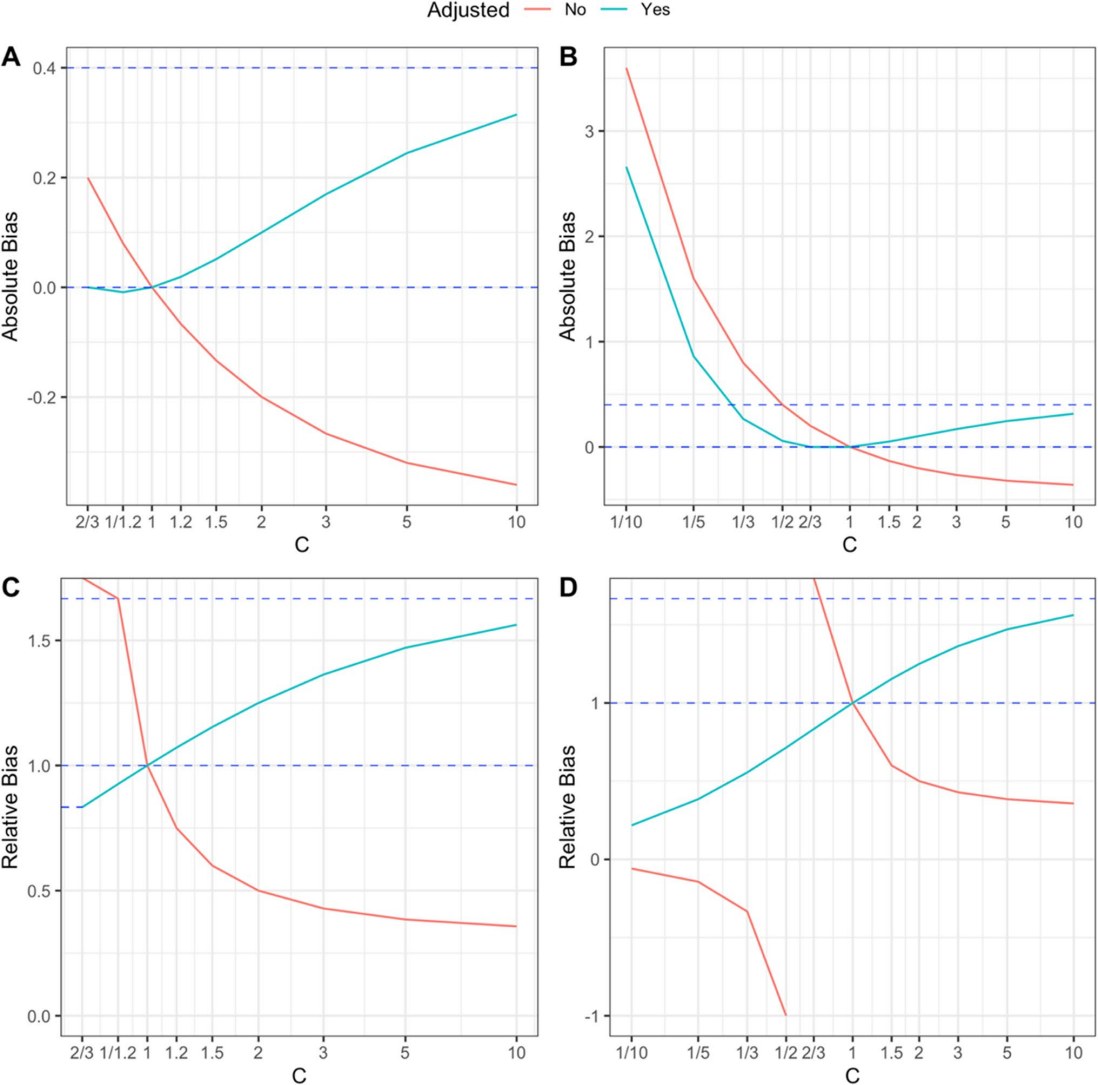
Plots for absolute and relative error in Levin’s formula when $R{R}_{U}=1.5$ and $\pi =0.5$ over a range for the confounding ratio $C$, assuming no effect-size modification on the relative risk scale. **A**: absolute bias, $R{R}_{C}\ge 1$, **B**: absolute bias, no restriction on $R{R}_{C}$, **C**: relative bias, $R{R}_{C}\ge 1$, D: relative bias, no restriction on $R{R}_{C}$. In the attached plots, the bias for the ‘adjusted’ version, $PA{F}_{L}=\frac{\pi (R{R}_{C}-1)}{1+\pi (R{R}_{C}-1)}$ of Levin’s formula is given in blue, while biases for the unadjusted version,$PA{F}_{L,U}=\frac{\pi (R{R}_{U}-1)}{1+\pi (R{R}_{U}-1)}$ are given in red. The upper blue horizontal lines are the limiting absolute bias: $1-\frac{\pi R{R}_{U}}{1+\pi (R{R}_{U}-1)}$ (for plots **A** and **B**) and relative bias: $1+\frac{1-\pi }{\pi R{R}_{U}}$ for $PA{F}_{L}$ as $C\to \infty$ (for plots **C** and **D**). Note that the absolute bias of $PA{F}_{L}$ is 0 at both $C=1/R{R}_{U}$ and $C=1$, while the relative bias for $PA{F}_{L}$ converges to $\frac{1+\pi (R{R}_{U}-1)}{R{R}_{U}}$ as $C$ approaches $1/R{R}_{U}$ (as tagged on the y-axis of plot **C**). The absolute and relative biases of the unadjusted version of Levin’s formula are more erratic. In particular, the relative bias function for $PA{F}_{L,U}$ is negative when $R{R}_{C}<1$ or equivalently when $C<\frac{1}{R{R}_{U}}$ indicating that $PA{F}_{L,U}$ shows the incorrect direction of association in this scenario; in contrast the relative bias for $PA{F}_{L}$ is always larger than 0 for all $C>0$ and converges to 0 as $C\to 0$

In supplementary section S8, we analyse Eq. (5) mathematically, and show this relative bias function gets worse as the degree of confounding increases in either direction. $\frac{PA{F}_{L}}{PAF}$ is 1, when there is no confounding, that is when *C* = 1*.* Fixing the prevalence and unadjusted relative risk, $\frac{PA{F}_{L}}{PAF}$ increases to a maximum of $1+\frac{1-\pi }{\pi R{R}_{U}}>1$ as the confounding ratio *C* increases, and decreases to 0 when *C* approaches 0. The absolute bias, $PA{F}_{L}-PAF$, is again 0 when there is no confounding, that is when $C=1$, or when there is no causal effect of the risk factor, $R{R}_{C}=1$, and increases to $1-\frac{\pi R{R}_{U}}{1+\pi (R{R}_{U}-1)}$ for large *C*. One can also prove that the relative bias, (5), will get worse as the risk factor prevalence, $\pi ,$ gets smaller, although the relationship between $\pi$ and absolute bias is more complicated (absolute bias approaches 0 when $\pi$ is close to 0 and when $\pi$ is close to 1, fixing ${RR}_{U}$ and *C*). These results (and other properties of the relative and absolute bias functions) are proven in Supplementary Section S8. A graphical representation of the associated biases (similar to the plots in [10], which also includes a representation of limiting biases and behaviour in the ‘unadjusted’ version of Levin’s formula, (2), using $R{R}_{U}$ in place of $R{R}_{C}$ is given by Fig. 1.

## PAF for exposures with more general distributions

When the distribution of exposure is multi-category or continuous, PAF can be defined as the fraction of prevalent (or incident) disease cases that would have been avoided in a population where the value of the exposure was fixed at a particular value or follows a distribution that minimises the probability of disease (this value is known as the minimum risk exposure value or MREV). While with individual-level data it is possible to estimate the MREV [16], when deriving estimates of PAF from summary estimates one usually assumes a pre-determined MREV [6]. For example, in the simple case of an exposure such as air pollution which can be eliminated,^4^ the MREV would be presumed to be 0, that is elimination, with non-zero values being appropriate for exposures like blood pressure or sodium consumption. In the following, we will assume for simplicity that the MREV = 0, so that PAF is still defined by (1). The causal relative risk is now a function, $R{R}_{C}\left(x\right)=\frac{P({Y}_{x}=1)}{P({Y}_{0}=1)}$, comparing the increased prevalence of disease if the population were all exposed to exposure level $x$ relative to disease prevalence if the same were all exposed to the MREV. Miettinen’s formula (4) can be extended as follows:

6 $$PA{F}_{M}={E}_{X|Y=1}\left[\frac{R{R}_{C}(X)-1}{R{R}_{C}(X)}\right]$$

where ${E}_{X|Y=1}[f(X)]$ denotes the average of a function, $f(X)$, of the exposure, $X$ within the population of individuals with disease. Under the assumption that for each possible exposure value, $x,$ $R{R}_{C}(x)$ is constant within confounder strata, Eq. (6) will be correct for the PAF as given by (1), see supplementary section S5. Note that this formula is valid for both continuous exposures (such as air pollution) and multi-category exposures (such as a three-level coding of smoking in terms of current smokers/former smokers and never-smokers), and simplifies to (3) in the setting of a binary exposure.

As shown in supplementary section S7, (6) can be re-expressed using Bayes’ Rule as follows:

7 $$PA{F}_{M}=\frac{{E}_{X}\left(R{R}_{U}(X)\frac{R{R}_{C}(X)-1}{R{R}_{C}(X)}\right)}{{E}_{X}(R{R}_{U}(X))}=\frac{\int R{R}_{U}(x)\frac{R{R}_{C}(x)-1}{R{R}_{C}(x)}\pi (x)dx}{\int R{R}_{U}(x)\pi (x)dx},$$

with ${R}_{U}\left(x\right)=\frac{P(Y=1|X=x)}{P(Y=1|X=0)}$ representing the unadjusted relative risk function that compares the prevalence of disease in the strata of the population exposed to the value $x$ of the risk factor with the prevalence in the strata exposed to the MREV, and ${E}_{X}$ is the population expectation operator. The right-hand side of (7) indicates that one can calculate $PA{F}_{M}$ by numerical integration of the quantities $R{R}_{U}(x)\frac{R{R}_{C}(x)-1}{R{R}_{C}(x)}\pi (x)$ and $R{R}_{U}\left(x\right)\pi \left(x\right),$ where we are assuming that the exposure distribution is absolutely continuous with density $\pi (x)$. In the special case that the exposure is discrete, but with more than two levels: $X\in \{\mathrm{0,1},...,L\}$, with the population prevalences of each level: $\pi (0),...,\pi (L)$, and MREV = 0, Eq. (7) can be re-expressed as:

8 $$PA{F}_{M}=\frac{{\sum }_{x=1}^{x=L}\pi (x)R{R}_{U}(x)\frac{R{R}_{C}(x)-1}{R{R}_{C}(x)}}{\pi (0)+{\sum }_{x=1}^{x=l}\pi (x)R{R}_{U}(x)}.$$

## Bias from Levin’s approach for more general exposure distributions

The generalisation of Levin’s formula to continuous exposure distributions is given by:

9 $$PAF_{L} = \frac{{E_{X} \left( {RR_{C} \left( X \right)} \right) - 1}}{{E_{X} \left( {RR_{C} \left( X \right)} \right)}} = \frac{{\smallint RR_{C} \left( x \right)\pi \left( x \right)dx - 1}}{{\smallint RR_{C} \left( x \right)\pi \left( x \right)dx}}.$$

The formula for multi-category exposures is very almost identical, apart from replacing the integral in (9) with a summation over the values, $x$, that the exposure can take. See Table 2 for a comparison of Levin’s and Miettinen’s approach for the binary, multi-category and continuous exposures. Comparing Eqs. (7) and (9), it follows immediately that under no confounding, that is $R{R}_{U}(x)=R{R}_{C}(x)$ for every exposure value $x$, Levin’s formula is again unbiased. However, analysis of bias in Levin’s formula is more complicated when the distribution of the exposure is non-binary, although under certain special settings, one can analyse bias in the same way as in the binary exposure case. See supplementary section S9 for discussion.

**Table 2:** Below, we show the identification formulae for ${\mathrm{PAF}}$ and ${\mathrm{PAF}}_{\mathrm{L}}$, for binary, multicategory and continuous exposure distributions, under the assumption of no effect modification

	$PA{F}_{L}$	$PA{F}_{M}$ (in terms of ${\pi }_{c}$ and $R{R}_{C}$)	$PA{F}_{M}$ (in terms of $\pi$, $R{R}_{U}$ and $R{R}_{C}$)
Binary risk factor	$\frac{\pi (R{R}_{C}-1)}{1+\pi (R{R}_{C}-1)}$	${\pi }_{c}\frac{R{R}_{C}-1}{R{R}_{C}}$	$[\frac{\pi R{R}_{U}}{1+\pi (R{R}_{U}-1)}]\frac{R{R}_{C}-1}{R{R}_{C}}$
Multicategory risk factor	$\frac{{\sum }_{x=1}^{L}\pi (x)(R{R}_{C}(x)-1)}{1+{\sum }_{x=1}^{L}\pi (x)(R{R}_{C}(x)-1)}$	${\sum\nolimits_{l=1}^{L}}{\pi }_{c}(x)\frac{R{R}_{C}(x)-1}{R{R}_{C}(x)}$	$\frac{{\sum }_{x=1}^{x=L}\pi (x)R{R}_{U}(x)\frac{R{R}_{C}(x)-1}{R{R}_{C}(x)}}{\pi (0)+{\sum }_{x=1}^{x=L}\pi (x)R{R}_{U}(x)}$
Continuous risk factor	$\frac{\int R{R}_{C}(x)\pi (x)dx-1}{\int R{R}_{C}(x)\pi (x)dx}$	$\int \frac{R{R}_{C}(x)-1}{R{R}_{C}(x)}{\pi }_{c}(x)dx$	$\frac{\int R{R}_{U}(x)\frac{R{R}_{C}(x)-1}{R{R}_{C}(x)}\pi (x)dx}{\int R{R}_{U}(x)\pi (x)dx}$

$\uppi$ represents the population prevalence of a binary risk/ factor and ${\uppi }_{\mathrm{c}}$ the prevalence within disease cases, with similar notation representing probability mass functions and density functions when the exposure is multi-category or continuously distributed. An implicit assumption for the multicategory and continuous formulae is that the MREV (minimum risk exposure value) is equal to 0. In all settings, ${\mathrm{PAF}}$ is expressed using the traditional Miettinen formula (in terms of ${\uppi }_{\mathrm{c}}$ and ${\mathrm{RR}}_{\mathrm{C}}$) and the 3 variable formula advocated in this manuscript which can be used if estimates of both the unadjusted and adjusted relative risks are available

## Discussion

We use this final section mostly to discuss caveats and limitations to our suggested approaches. First, we have suggested that where possible Eq. (4) should be used instead of Levin’s formula, (2), to estimate PAF when individual-level data is unavailable. Equation (4) requires estimates of the unadjusted and causal relative risks, $\widehat{RR}_{U}$ and $\widehat{RR}_{C}$. In practice, relative risks are estimated in one population and used to estimate PAF in a differing population. This requires an implicit assumption that the relative risks are transportable across populations, that is they are equal in the population in which the relative risks are estimated and the population for which PAF is being estimated. While transportability of the causal relative risk is an issue both for Eq. (4) and Levin’s approach, (2), transportability of the unadjusted relative risk is an additional assumption and there is no good reason to expect it to hold.

Here we advocate that authors use Eq. (4) when estimates of unadjusted and adjusted relative risks are available. In contrast, in the literature we’ve noticed several examples of Levin’s formula being applied with unadjusted relative risks in observational settings [18, 19]. While this will generate correct results under no confounding, we would not advise this practice. First, if there is no confounding, either an adjusted or unadjusted relative risk would be appropriate to use in Levin’s approach, that is both should generate statistically consistent estimates of the true causal relative risk, provided no effect modifiers are included in the set of variables that are adjusted for in the adjusted relative risk. Second, in observational settings, there usually will be some degree of confounding in which case substituting unadjusted relative risks into Levin’s formula will often result in more egregious bias than if appropriately adjusted relative risks were instead used in the same formula. For example, using Levin’s formula with an unadjusted relative will flip the direction of the putative PAF when $R{R}_{U}<1$ and $R{R}_{C}>1$, so that while $PAF>0$ (the risk factor causes disease), $PA{F}_{L,U}=\frac{\pi \left(R{R}_{U}-1\right)}{1+\pi \left(R{R}_{U}-1\right)}<0$, that is the risk factor would seem to be protective when using an unadjusted relative risk with Levin’s approach. In contrast, this problem does not happen with using the true causal relative risk in Levin’s formula since $PA{F}_{L}=\frac{\pi \left(R{R}_{C}-1\right)}{1+\pi \left(R{R}_{C}-1\right)}>0$, when $R{R}_{C}>1$. Plots B and D in Fig. 1 show that, as one might expect, Levin’s formula also flips the direction of the causal effect if $R{R}_{U}>1$ and $R{R}_{C}<1$. In addition, for very small causal effects with $R{R}_{C}\approx 1$, $PA{F}_{L}\approx 0$ irrespective of the value of the confounding ratio $C$, however in the same scenario $PA{F}_{L,U}$ can be quite biased if $C$ is very large or very small.

As noted by other authors, the bias using Levin’s formula, with the causal relative risk, is generally quite small [10] and likely insubstantial compared to other biases involved in estimating a PAF. For instance, in an example regarding the relationship between physical inactivity and depression, there may be questions about the consistency of ascertainment and measurement of both the exposure (inactivity) and outcome (depression). The prevalence of defined inactivity (and the associated relative risk) will change depending on what the investigator considers to constitute inactivity, and the definition may differ across different studies. Similar issues arise in the consistency of ascertainment and measurement of depression. These issues reduce the real-world meaning and actionability of any estimated PAF. On a related point, physical activity should really be measured on a continuum; a binary definition of inactivity will likely underestimate associated disease burden. For instance, a better approach to calculating PAF might determine a sufficient level of physical activity (that perhaps differs dependent on age and other characteristics of a person) and estimate disease prevalence in a hypothetical population where everybody had at least this level of activity; however, implementation of such an estimator may be practically challenging. Finally, the assumption that the causal relative risk is constant across differing confounding strata will usually be dubious (individuals exposed or not exposed to differing patterns of confounding variables have differing baseline probabilities of disease making the same relative effect of a risk factor unlikely). If the causal relative risk varies over differing strata of confounders, formulae for $PAF$ for binary and general exposure distributions are as follows:

10 $$PAF={E}_{C|Y=1}[P(X=1|C,Y=1)\frac{R{R}_{C}(C)-1}{R{R}_{C}(C)}]$$

11 $$PAF={E}_{C|Y=1}\left[{E}_{X|C,Y=1}\left[\frac{R{R}_{C}\left(X,C\right)-1}{R{R}_{C}\left(X,C\right)}\right]\right],$$

where $R{R}_{C}\left(c\right)=\frac{P({Y}_{1}=1|C=c)}{P({Y}_{0}=1|C=c)}$ is regarded as the causal relative risk within confounder stratum $C=c$ in (10) and $R{R}_{C}\left(x,c\right)=\frac{P({Y}_{x}=1|C=c)}{P({Y}_{MREV}=1|C=c)}$ as the causal relative risk comparing exposure levels $x$ and the MREV in confounder stratum $C=c$ in (11). Note again that (10) can be re-expressed as: $PAF=P(X=1|Y=1)\frac{R{R}_{e}-1}{R{R}_{e}}$, where $R{R}_{e}=\frac{P({Y}_{1}=1|X=1)}{P({Y}_{0}=1|X=1)}$ is the causal relative risk restricted to the population exposed to the risk factor, as Miettinen originally showed in 1972. These formulae are derived in supplementary sections S2 and S6 and are equivalent to, and extend of in the case of (11), the formulae suggested in [17], and without a no-effect modification assumption require individual-level data (incorporating effect modification between risk factors and confounders) to estimate. When relative risks vary over confounder strata, the marginal causal relative risk, $R{R}_{C}$ (defined as the ratio of disease probabilities if everyone was exposed and everyone was exposed to the risk factor), and the causal relative risk in exposed individuals are both weighted average of confounder-strata specific causal relative risks, $R{R}_{C}(c)$ (see Supplementary Appendix S10). As a result, one would not expect large differences between (4) and (10) under moderate levels of effect modification.

While equivalent results to Eq. (4) exist for multi-category and continuous exposures (Eqs. (7)–(8)) these may be not as useful in practice as Eq. (4) as they require specification of unadjusted relative risks comparing many levels of exposure to baseline. As a result, Levin’s formula (despite its bias) may forever be the method of choice for estimating PAFs and impact fractions for continuous exposures with summary data. However, the biases in Levin’s formulae are often dismissed in this setting. If the formula is to be used it is important to recognise its bias and have some awareness of the likely extent of the bias. In this regard, if there is a range of values of the exposure which approximately minimises disease risk and it’s plausible that the confounding parameter: $C(X)=\frac{R{R}_{C}(X)}{R{R}_{U}(X)}$ is approximately constant outside of this range, Eq. (2) in supplementary section S9 may be useful in determining the likely error from using Levin’s approach.

### Supplementary Information

Below is the link to the electronic supplementary material.

Supplementary file1 (PDF 282 kb)

Supplementary file2 (XLSX 10 kb)

## Footnotes

1 Note that if *Y* is an indicator for incident disease over a period of time, rather than prevalent disease, in a cohort of healthy individuals at baseline, the PAF as defined by (1) would be interpreted as the proportion of incident disease in the cohort over that period of time that would have been avoided if the entire cohort were risk-factor unexposed. For simplicity, in this manuscript we will interpret PAFs in terms of decreases in prevalent, rather than incident, disease.

2 $\widehat{R{R}_{C}}$ may be biased for the true causal relative risk, $R{R}_{C}$, due to residual confounding. However, a discussion of ensuing bias in the estimated PAF due to residual confounding is beyond the scope of this manuscript, where we are solely concerned with the difference in the estimands $PA{F}_{L}$ and $PA{F}_{M}$.

3 Note that (5) is technically the relative discrepancy (or error) between the estimands $PA{F}_{L}$ and $PAF$. However, if statistically consistent estimates of $R{R}_{C}$ and $\pi$ are plugged in to Levin’s formula, (5) will represent the asymptotic bias of the resulting estimator. As a result, we will (somewhat informally) refer to (5) and comparable quantities as biases throughout the manuscript.

4 Being a measure of the total disease burden attributable to an exposure or risk factor, defining PAF necessitates consideration of hypothetical populations (such as a world without air pollution) that may be unachievable under realistic public health interventions. This distinguishes PAF from the related idea of impact fractions which can be loosely defined as the relative decrease in disease prevalence achievable from a practically implementable intervention on a risk factor.
