# Supplementary material for: Bias assessment and correction for Levin’s population attributable fraction in the presence of confounding
Source: Eur J Epidemiol. 2024 Jan 3;39(2):111–9. doi: 10.1007/s10654-023-01063-8 (PMC10904572; doi:10.1007/s10654-023-01063-8)
Supplement: Supplementary file 1 — Supplementary file1 (PDF 282 kb) [file 10654_2023_1063_MOESM1_ESM.pdf]

Supplementary Material

S1A Proof that $PAF = \pi_c \frac{RR_C - 1}{RR_C}$, where $\pi_c = P(X = 1 \mid Y = 1)$ and $RR_C = \frac{P(Y_1 = 1)}{P(Y_0 = 1)}$ provided that:

1) Conditional exchangeability: $Y_x \perp X \mid C = c$, holds within each confounder strata c, for $x \in \{0,1\}$[1]
2) Consistency: If $X = x$, $Y = Y_x$, for $x \in \{0,1\}$
3) $RR_C(c) = \frac{P(Y = 1 \mid X = 1,c)}{P(Y = 1 \mid X = 0,c)} = K$[2] is constant over confounder strata $C = c$

Proof

First, we note that:

$$P(Y = 1 \mid X = x, c) = P(Y_x = 1 \mid X = 1,c) = P(Y_x = 1 \mid c), x \in \{0,1\}$$

with the first equality due to consistency and the second due to assumption 1) above. This indicates that:

$$RR_C(c) = \frac{P(Y = 1 \mid X = 1,c)}{P(Y = 1 \mid X = 0,c)} = \frac{P(Y_1 = 1 \mid c)}{P(Y_0 = 1 \mid c)} = K$$

that is the ratio of conditional probabilities of disease under exposure and non-exposure in stratum $C = c$ equates to the causal relative risk in stratum c, which we assume is a constant K

Now note that

[1] Technically 'mean' conditional exchangeability, that is $P(Y_a^* = 1 \mid X = a, C = c) = P(Y_a^* = 1 \mid C = c)$ for any values $\{a, a^*\} \in \{0,1\}^2$ is sufficient

[2] Note, in the proof below and subsequent proofs, we utilise upper case letters to represent random variables and lower case letters to represent values those random variables may take. We assume for simplicity that the distribution of confounders is discrete, so that we can represent expectations of functions of confounder variables over the population: $E_C f(C)$ as a sum over differing values $\sum_c f(c)P(c)$, with $P(c)$ is shorthand for $P(C = c)$. We also abbreviate conditional expectations of confounder variables such as: $P(X = 1 \mid C = c)$ as $P(X = 1 \mid c)$.

$$RR_C = \frac{P(Y_1 = 1)}{P(Y_0 = 1)} = \frac{E(P(Y_1 = 1 | C))}{E(P(Y_0 = 1 | C))} = \frac{E(P(Y_0 = 1 | C)\frac{P(Y_1 = 1 | C)}{P(Y_0 = 1 | C)})}{E(P(Y_0 = 1 | C))} = K$$

indicating that $RR_C(c) = RR_C$ for all confounder strata c

The proof proceeds as follows:

$$PAF = \frac{P(Y = 1) - P(Y_0 = 1)}{P(Y = 1)}$$

$$= \frac{P(Y = 1) - E_C(P(Y_0 = 1 | C))}{P(Y = 1)}$$

$$= \frac{P(Y = 1) - E_C(P(Y_0 = 1 | X = 0, C))}{P(Y = 1)} \text{ (by assumption 1)}$$

$$= \frac{P(Y = 1) - E_C(P(Y = 1 | X = 0, C))}{P(Y = 1)} \text{ (by consistency)}$$

$$= \frac{E_{X,C}(P(Y = 1 | X, C)) - E_C(P(Y = 1 | X = 0, C))}{P(Y = 1)}$$

$$= \frac{E_C(P(X = 1 | C)P(Y = 1 | X = 1, C) + P(X = 0 | C)P(Y = 1 | X = 0, C)) - E_C(P(Y = 1 | X = 0, C))}{P(Y = 1)}$$

$$= \frac{E_C(P(X = 1 | C)P(Y = 1 | X = 1, C) + P(X = 0 | C)P(Y = 1 | X = 0, C) - P(Y = 1 | X = 0, C))}{P(Y = 1)}$$

$$= \frac{E_C(P(X = 1 | C)P(Y = 1 | X = 1, C) - P(X = 1 | C)P(Y = 1 | X = 0, C))^{*}}{P(Y = 1)}$$

$$= (RR_C - 1)\frac{E_C(P(X = 1 | C)P(Y = 1 | X = 0, C))}{P(Y = 1)} \text{(since } RR_C = \frac{P(Y = 1 | X = 1, C)}{P(Y = 1 | X = 0, C)}\text{)}$$

$$= \frac{(RR_C - 1)}{RR_C}\frac{E_C(P(X = 1 | C)P(Y = 1 | X = 1, C))}{P(Y = 1)} \text{(since } RR_C^{-1} = \frac{P(Y = 1 | X = 0, C)}{P(Y = 1 | X = 1, C)}\text{)}$$

$$= \frac{P(X = 1, Y = 1)}{P(Y = 1)} \times \frac{RR_C - 1}{RR_C} \text{ (since } P(X = 1, Y = 1) = E_C(P(X = 1, Y = 1 | C))\text{)}$$

$$= P(X = 1 | Y = 1) \times \frac{RR_C - 1}{RR_C}$$

$$= \pi_c \frac{RR_C - 1}{RR_C}$$

S1B. Proof that $PAF = \pi \frac{RR_U - 1}{1 + \pi(RR_U - 1)}$ under

1) Marginal exchangeability: $Y_x \perp X$ for $x \in \{0,1\}$
2) Consistency: If $X = x$, $Y = Y_x$, for $x \in \{0,1\}$

Proof

Under the above marginal exchangeability assumption, one can show equality of the causal and unadjusted relative risks:

$$RR_C = \frac{P(Y_1 = 1)}{P(Y_0 = 1)} = \frac{P(Y_1 = 1 \,|\, X = 1)}{P(Y_0 = 1 \,|\, X = 0)} = \frac{P(Y = 1 \,|\, X = 1)}{P(Y = 1 \,|\, X = 0)} = RR_U$$

where assumption 1 is necessary for the first equality and assumption 2 for the second equality.

$$PAF = \frac{P(Y = 1) - P(Y_0 = 1)}{P(Y = 1)} = \frac{P(Y = 1) - P(Y = 1 \,|\, X = 0)}{P(Y = 1)} \text{ (assumptions 1 and 2)}$$

$$= \frac{P(Y = 1 \,|\, X = 0)P(X = 0) + P(Y = 1 \,|\, X = 1)P(X = 1) - P(Y = 1 \,|\, X = 0)}{P(Y = 1)}$$

$$= \frac{P(X = 1)(P(Y = 1 \,|\, X = 1) - P(Y = 1 \,|\, X = 0))}{P(Y = 1)}$$

$$= \frac{P(X = 1)P(Y = 1 \,|\, X = 1)(1 - RR_U^{-1})}{P(Y = 1)} = \pi_c(1 - RR_U^{-1})$$

An application of Bayes' Rule (which here will demonstrate that: $\pi_c = \frac{\pi RR_U}{1 + \pi(RR_U - 1)}$, see Section 3 of the Supplementary material) then shows that the above expression equals:

$$PAF = \frac{\pi RR_U}{1 + \pi(RR_U - 1)}(1 - RR_U^{-1}) = \frac{\pi(RR_U - 1)}{1 + \pi(RR_U - 1)}$$

Since we have also shown $RR_U = RR_C$, this can also be expressed as:

$$PAF = \frac{\pi(RR_C - 1)}{1 + \pi(RR_C - 1)}$$

Note that no assumptions regarding effect modification were necessary in the above derivation.

Section 2

Expressions for Miettinen's formula under effect modification

In this section, we give proofs of more general formulations of Miettinen's formula. We assume 1) and 2) from Section S1, but now allow the causal relative risk: $RR_C(c)$ to vary over covariate strata c.

S2A Expression for Miettenin's PAF when the causal relative risk $RR_C(c)$ varies over confounder strata:

First we show that:

$$PAF = E_{C|Y=1}(P(X=1|Y=1,C)\frac{RR_C(C)-1}{RR_C(C)})$$

where $RR_C(c) = \frac{P(Y_1 = 1|C=c)}{P(Y_0 = 1|C=c)}$ is the causal relative risk within stratum $C = c$.

Proof:

The steps to show this result are identical to as in Supplementary Section 1A, up to the asterisk in that proof. The proof proceeds as follows

$$PAF = \frac{E_C(P(Y=1,X=1|C)\frac{RR_C(C)-1}{RR_C(C)})}{P(Y=1)}$$

$$= \frac{E_C(P(Y=1|C)P(X=1|Y=1,C)\frac{RR_C(C)-1}{RR_C(C)})}{P(Y=1)}$$

$$= \frac{\sum_c P(c)P(Y=1|C)P(X=1|Y=1,C)\frac{RR(c)-1}{RR(c)}}{P(Y=1)}$$

$$= \sum_c P(c|Y=1)P(X=1|Y=1,C)\frac{RR(c)-1}{RR(c)}$$

$$= E_{C|Y=1}(P(X=1|Y=1,C)\frac{RR_C(C)-1}{RR_C(C)}) \quad (1)$$

When individual data $(c_i, x_i, y_i)_{i \le N}$ is available from the population of interest, the above formula: $PAF = E_{C|Y=1}(P(X=1 \mid Y=1,C)\frac{RR_C(C)-1}{RR_C(C)})$ can re be used to estimate PAF by averaging $I\{x_i = 1\}\frac{\hat{RR}_C(c_i)-1}{\hat{RR}_C(c_i)}$ over the subset of cases in the data: $\{i \le N : y_i = 1\}$ where $\hat{RR}_C(c_i)$ is the estimated relative risk of disease for an individual with covariates c_i. Regression type methods can facilitate estimation of $\hat{RR}_C(c_i)$.

S2B Original formulation for Miettinen's formula:

Here we will show that:

$$PAF = P(X=1 \mid Y=1)\frac{RR_e - 1}{RR_e}$$

where RR_e is the causal relate risk: $\frac{P(Y_1 = 1 \mid X=1)}{P(Y_0 = 1 \mid X=1)}$ in the subpopulation exposed to the risk factor.

Proof

We start with the expression for PAF that was proven in S2A

$$PAF = E_{C|Y=1}(P(X=1 \mid Y=1,C)\frac{RR_C(C)-1}{RR_C(C)})$$

$$= \sum_c P(c \mid Y=1)P(X=1 \mid Y=1,c)\frac{RR_C(c)-1}{RR_C(c)} \qquad (2)$$

Since

$$P(X=1 \mid Y=1,c) = P(Y=1)P(X=1 \mid Y=1)P(c \mid X=1,Y=1)/P(c)P(Y=1 \mid c)$$

and $P(c \mid Y=1) = P(c)P(Y=1 \mid c)/P(Y=1)$, (2) equals:

$$P(X=1 \mid Y=1) \sum_c P(c \mid X=1, Y=1) \frac{RR_C(c) - 1}{RR_C(c)} \quad (3)$$

Noting $\frac{RR_c(c) - 1}{RR_c(c)} = \frac{P(Y=1 \mid X=1, c) - P(Y=1 \mid X=0, c)}{P(Y=1 \mid X=1, c)}$ and

$p(c \mid X=1, Y=1) = P(Y=1 \mid X=1, c)P(c)P(X=1 \mid c)/P(X=1)P(Y=1 \mid X=1)$,
it follows that the above display is equal to

$$P(X=1 \mid Y=1) \sum_c \frac{P(c)P(X=1 \mid c)[P(Y=1 \mid X=1, c) - P(Y=1 \mid X=0, c)]}{P(X=1)P(Y=1 \mid X=1)} \quad (4)$$

Noting:
$P(c)P(X=1 \mid c)/\{P(X=1)P(Y=1 \mid X=1)\} = p(c \mid X=1)/P(Y=1 \mid X=1)$

and $\sum_c P(c \mid X=1)P(Y=1 \mid X=1, c) = P(Y=1 \mid X=1)$, the above equals:

$$P(X=1 \mid Y=1) \frac{P(Y=1 \mid X=1, c) - \sum_c P(c \mid X=1)P(Y=1 \mid X=0, c)}{P(Y=1 \mid X=1)} \quad (5)$$

Finally, letting the causal relative risk in risk in the exposed be:
$RR_e = \frac{P(Y_1=1 \mid X=1)}{P(Y_0=1 \mid X=1)} = \frac{P(Y=1 \mid X=1)}{E_{C|X=1}P(Y_0=1 \mid X=1, C)} = \frac{P(Y=1 \mid X=1)}{E_{C|X=1}P(Y=1 \mid X=0, C)}$

where the first equality assumes consistency and the second equality assumes conditional exchangeability and consistency , it follows that in general PAF can be expressed as:

$$P(X=1 \mid Y=1) \frac{RR_e - 1}{RR_e} \quad (6)$$

S3. Proof that under the assumptions of S1, $PAF = \frac{\pi RR_U}{1+\pi(RR_U-1)}\frac{RR_C-1}{RR_C}$

By S1A, the result will be true if $\pi_c = \frac{\pi RR_U}{1+\pi(RR_U-1)}$; this can be proven using Bayes' Rule.

Proof:

$$
\begin{aligned}
\pi_c &= P(X=1\,|\,Y=1) \\
&= \frac{P(X=1,Y=1)}{P(Y=1)} \\
&= \pi\frac{P(Y=1\,|\,X=1)}{\pi P(Y=1\,|\,X=1)+(1-\pi)P(Y=1\,|\,X=0)} \\
&= \pi\frac{1}{\pi+(1-\pi)RR_U^{-1}} \\
&= \frac{\pi RR_U}{1+\pi(RR_U-1)}
\end{aligned}
$$

This implies that:

$$PAF = [\frac{\pi RR_U}{1+\pi(RR_U-1)}]\frac{RR_C-1}{RR_C}$$

S4. Derivation of Formula (5) in the main manuscript for relative bias under the assumptions in S1

Remembering that $PAF_L = \pi \dfrac{RR_C - 1}{1 + \pi(RR_C - 1)}$ and under the assumptions in S1,

$PAF = [\dfrac{\pi RR_U}{1 + \pi(RR_U - 1)}]\dfrac{RR_C - 1}{RR_C}$, it follows that:

$$B = \frac{PAF_L}{PAF}$$

$$= \frac{(RR_C - 1)(1 + \pi(RR_U - 1))}{RR_U(1 + \pi(RR_C - 1))} \times \frac{RR_C}{RR_C - 1}$$

$$= \frac{1 + \pi(RR_U - 1)}{1 + \pi(RR_C - 1)} \times \frac{RR_C}{RR_U}$$

$$= \frac{1 + \pi(RR_U - 1)}{1 + \pi(C \times RR_U - 1)} \times C$$

S5. Generalisation of Miettinen's formula for continuous (or multi-category) exposure distributions

For a strata of confounders, $C = c$ we will assume that $RR(x,c) = \dfrac{P(Y=1\,|\,X=x, C=c)}{P(Y=1\,|\,X=0, C=c)} = K_x$ is constant over all confounder strata $C = c$ for each exposure value x.

We assume without loss of generality, that the Minimum risk exposure value is 0 (MREV = 0), and write, $RR_C(x) = \dfrac{P(Y_x = 1)}{P(Y_0 = 1)}$ for the causal relative risk at exposure value $X = x$, relative to the MREV. Note this is somewhat an abuse of notation since in section S3, we used $RR_C(c)$ to refer to the causal relative risk in confounder stratum $C = c$. In practice, we use context to distinguish $RR_C(c)$ and $RR_C(x)$, with the convention that we always use fixed c to represent confounder strata and fixed x to represent an exposure value.

Below, we also assume conditions 1 and 2 (conditional exchangeability and consistency) given in S1, but now relative to the continuously distributed exposure X; previously the implicit assumption was a binary exposure.

Finally, note the symbol π is used to denote a generic density function for the exposure X, with its arguments determining the precise density or conditional specified.

Proof

Note similarly to in S1:

$$RR(c,x) = \frac{P(Y=1\,|\,X=x, c)}{P(Y=1\,|\,X=0, c)} = \frac{P(Y_1 = x\,|\,c)}{P(Y_0 = 1\,|\,c)} = K_x$$

where the second equality follows by using both consistency and conditional exchangeability assumptions/

which implies that, for each fixed value x

$$RR_C(x) = \frac{P(Y_1 = 1 \mid X = x)}{P(Y_0 = 1 \mid X = x)} = \frac{E_C(P(Y_1 = 1 \mid C, X = x))}{E_C(P(Y_0 = 1 \mid C, X = x))}$$

$$= \frac{E_C(P(Y_0 = 1 \mid C, X = x)\frac{P(Y_1 = 1 \mid C, X = x)}{P(Y_0 = 1 \mid C, X = x)})}{E_C(P(Y_0 = 1 \mid C, X = x))} = K_x$$

so that $\frac{P(Y = 1 \mid X = x, c)}{P(Y = 1 \mid X = 0, c)} = RR_C(x)$

It then follows that:

$$PAF = \frac{P(Y = 1) - P(Y_0 = 1)}{P(Y = 1)}$$

$$= \frac{P(Y = 1) - E_C(P(Y = 1 \mid X = 0, C))}{P(Y = 1)}$$

$$= \frac{E_{X,C}(P(Y = 1 \mid X, C)) - E_C(P(Y = 1 \mid X = 0, C))}{P(Y = 1)}$$

$$= \frac{E_C(\int \pi(x \mid C)(P(Y = 1 \mid X = x, C) - P(Y = 1 \mid X = 0, C))dx)}{P(Y = 1)}$$

$$= \frac{E_C(\int \pi(x \mid C)\frac{RR(x, C) - 1}{R(x, C)}P(Y = 1 \mid X = x, C)dx)}{P(Y = 1)}$$

$$= \frac{\sum_c P(c)(\int \pi(x \mid c)\frac{RR(x, c) - 1}{R(x, c)}P(Y = 1 \mid X = x, c)dx)}{P(Y = 1)}$$

Now noting that:

$$\pi(x \mid c, Y = 1) = \frac{P(Y = 1 \mid X = x, c)P(c)\pi(x \mid c)}{P(c)P(Y = 1 \mid c)} = \frac{P(Y = 1 \mid X = x, c)\pi(x \mid c)}{P(Y = 1 \mid c)}$$

so that

$$\frac{P(c)\pi(x \mid c)P(Y=1 \mid X=x,c)}{P(Y=1)} = \frac{\pi(x \mid c, Y=1)P(c)P(Y=1 \mid c)}{P(Y=1)} = \pi(x \mid c, Y=1)P(c \mid Y=1)$$

, the previous displayed equation must equal:

$$= \sum_c P(c \mid Y=1)[\int \pi(x \mid C, Y=1)\frac{RR(x,c)-1}{RR(x,c)}dx]$$

$$= \int \sum_c [P(c \mid Y=1)\pi(x \mid c, Y=1)\frac{RR_C(x)-1}{RR_C(x)}]dx$$

$$= \int \pi(x \mid Y=1)\frac{RR_C(x)-1}{RR_C(x)}dx \quad (1)$$

$$= E_{X \mid Y=1}[\frac{RR_C(X)-1}{RR_C(X)}]$$

With the second last line using Fubini's theorem to reverse the order of integration, the iterated expectation $E_{C \mid Y=1}\pi(x \mid C, Y=1) = \sum_c P(c \mid Y=1)\pi(x \mid c, Y=1) = \pi(x \mid Y=1)$ and

$RR(x,c) = RR_C(x)$.

S6. Proof of generalisation of Miettinen's formula for continuous (or multi-category) exposure distributions when causal relative risks vary over confounder strata:

The proof is essentially the same as the preceding proof. Note the penultimate line before equation (1) in S5 can be re-expressed as:

$$E_{C|Y=1}(\int \pi(x\,|\,C, Y = 1)\frac{RR(x,c) - 1}{RR(x,c)}dx = E_{C|Y=1}[E_{X|C,Y=1}[\frac{RR(X,C) - 1}{RR(X,C)}]]$$

which is the formula in question.

S7. Proof of formula (7)

$$
\begin{aligned}
&\pi(x \mid Y=1) \\
&= \frac{\pi(x)P(Y=1 \mid x)}{\int_x \pi(x)P(Y=1 \mid x)dx} \text{ (Bayes' Rule)} \\
&= \frac{\pi(x)RR_U(x)}{\int \pi(x)RR_U(x)dx} \text{ (Divide above and below by } P(Y=1 \mid 0)) \\
&= \frac{\pi(x)RR_U(x)}{E_X(RR_U(X))}
\end{aligned}
$$

Plugging the above expression into equation (1) in section S5 gives:

$$
PAF = \int \frac{\pi(x)RR_U(x)}{E_X(RR_U(X))} \frac{RR_C(x)-1}{RR_C(x)} dx = \frac{E_X(RR_U(X)\frac{RR_C(X)-1}{RR_C(X)})}{E_X(RR_U(X))}
$$

S8. Analysis of the derivatives of the Relative Bias equation, (5), in the main manuscript.

The derivitives with respect to C and π are as follows:

$$\frac{d}{dC}\frac{1+\pi(RR_U-1)}{1+\pi(C\times RR_U-1)}\times C$$

$$=\frac{(1+\pi(RR_U-1))(1+\pi(C\times RR_U-1))-C\pi RR_U(1+\pi(RR_U-1))}{(1+\pi(C\times RR_U-1))^2}$$

$$=\frac{(1+\pi(RR_U-1))(1+\pi(C\times RR_U-1)-C\pi RR_U)}{(1+\pi(C\times RR_U-1))^2}$$

$$=\frac{(1+\pi(RR_U-1))(1-\pi)}{(1+\pi(C\times RR_U-1))^2}>\frac{(1-\pi)^2}{(1+\pi(C\times RR_U-1))^2}>0 \quad \text{for all C}$$

$$\frac{d}{d\pi}\frac{1+\pi(RR_U-1)}{1+\pi(C\times RR_U-1)}\times C$$

$$=\frac{-(1+\pi(RR_U-1))(C\times RR_U-1))+(1+\pi(C\times RR_U-1))(RR_U-1)}{(1+\pi(C\times RR_u-1))^2}\times C$$

$$=\frac{RR_U(1-C)}{(1+\pi(C\times RR_u-1))^2}\times C$$

This derivative is negative for $C>1$ and positive for $C<1$

(In each case, smaller prevalences increase relative biases - but should decrease absolute biases)

Note that:

As $C \to \infty$, $\frac{1+\pi(RR_U - 1)}{1+\pi(C \times RR_U - 1)} \times C \to 1 + \frac{(1-\pi)}{\pi RR_U}$

As $C \to 0$, $\frac{1+\pi(RR_U - 1)}{1+\pi(C \times RR_U - 1)} \times C \to 0$

Bias in Levin's formula when $C > 1$

For values of $C > 1$, Levin's formula is biased above with the relative bias increasing toward a limit of $1 + \frac{1-\pi}{\pi RR_U}$ as $C \to \infty$ (note that this analysis assumes π and RR_U are fixed). As one would expect under no confounding in risk ratio, $C = 1, \frac{PAF_L}{PAF} = 1$ (that is the Levin estimand and true PAF are equal). Provided $RR_U > 1$, the absolute bias: $PAF_L - PAF$ also increases as C increases (from $PAF_L - PAF = 0$ when $C = 1$ to $1 - \frac{\pi RR_U}{1+\pi(RR_U - 1)}$ as $C \to \infty$). Note that Levin's formula, (2) in the main manuscript, converges to 1 as $C \to \infty$ irrespective of the values of π and RR_U, whereas the true PAF converges to $\frac{\pi RR_U}{1+\pi(RR_U - 1)}$ as C gets very large. In practice, imagining what the bias might be for extremely large values of C is interesting theoretically in it demonstrates the maximum possible bias in Levin's approach; but whether one would see such extreme confounding in a real example is debatable. For instance, a scenario where extreme values of C is possible is if there is a second risk factor X^* that acts as a confounder for $X \to Y$, with a strong causal influence on X (so much so that $X^* \sim X$ in the population), and such that the direct effect of X^* (not mediated through X) is to

strongly reduce the likelihood of disease (by a factor of $\frac{1}{C}$) but the effect of X on Y is to strongly increase the likelihood of disease by factor of C. Such scenarios are likely implausible in practical situations.

When $C > 1$, $\frac{\delta}{\delta\pi}\frac{1+\pi(RR_u-1)}{1+\pi(C \times RR_u - 1)} \times C < 0$ (fixing RR_U and C), indicating that as risk factor prevalence, π, decreases risk factor relative bias, increases. Given that relative bias is larger than 1 when $C > 1$, this indicates that relative bias worsens as risk factor prevalence gets smaller, although the situation for the degree of absolute bias is more complicated (absolute bias will converge to 0 as $\pi \downarrow 0$ and as $\pi \uparrow 1$).

Bias in Levin's formula when $C \leq 1$

For $C < 1$, Levin's formula is biased below and the relative bias becomes progressively worse, eventually converging towards 0 as $C \downarrow 0$ (a realm in which risk factors are protective and attributable fractions are negative). However, usually we imagine a risk factor coding such that $RR_C \geq 1$. Assuming that $RR_U > 1$, the absolute bias $PAF_L - PAF$ is 0 under no causal effect, $RR_C = 1$, (or equivalently for $C = \frac{1}{RR_U}$) in addition to being 0 when $C = 1$ (that is under no confounding). By continuity of the expressions (2), (3) and (4) in the main manuscript as functions of C we can argue that when the true causal effect is very small, $RR_C \approx 1$, the absolute bias in Levin's formula will be negligible, while the relative bias will be approximately $\frac{1+\pi(RR_U-1)}{RR_U} < 1$, although arguably the relative bias is not so unimportant in this case. The lesson here is that Levin's formula can be appropriate to use in the scenario that the true causal effect is very small or when there is no confounding).

When $C < 1$, $\frac{\delta}{\delta\pi}\frac{1+\pi(RR_u - 1)}{1+\pi(C \times RR_u - 1)} \times C > 0$ (fixing RR_U and C), indicating that as risk prevalence decreases, relative bias decreases. Given that the relative bias function is smaller than 1 when $C < 1$, this indicates again that relative bias gets worse as risk factor prevalence gets smaller, although as in the situation where $C > 1$ absolute bias converges to 0 as π converges to 0 or as π converges to 1.

S9. Expression for relative bias under more general exposure distributions:

To make progress here, suppose that the $MREV = 0$ and the prevalence of the minimum risk exposure level in the general population is $1 - \pi$ where $1 > 1 - \pi > 0$. We will also assume that the confounding ratio $C(X) = \frac{R_C(X)}{R_U(X)} = C$ at all exposure levels not equal to the MREV. In this case, we show below that the relative bias in the Levin estimate is:

(1) $$\frac{PAF_L}{PAF} = \frac{1 + \pi(E_{X|X>0}RR_U(X) - 1)}{1 + \pi(C \times E_{X|X>0}RR_U(X) - 1)} \times C,$$

, an expression very similar to equation (5) in the main manuscript, and which indicates that larger relative biases are expected under larger levels of confounding (that is $C < < 1$ or $C > > 1$). For continuous exposures, the assumption that a non-zero proportion of the population is exposed to the minimum exposure level may well be implausible. However a similar equation to (1) will hold appropriately if there is a range of exposure values, R, having non zero probability in the population which approximately minimise counterfactual risk: $\max_{x \in R} RR_C(x) \approx 1$, where $0 < \pi = P(X \notin R) < 1$ and $C(x) = \frac{R_C(x)}{R_U(x)} = C$ when $x \notin R$. The modified equation then would be:

(2) $$\frac{PAF_L}{PAF} \sim \frac{1 + \pi(E_{X|X \notin R}RR_U(X) - 1)}{1 + \pi(C \times E_{X|X \notin R}RR_U(X) - 1)} \times C.$$

Lemma. Suppose X is a positive continuous exposure with $MREV = 0$, where $0 < 1 - \pi = P(X = 0) < 1$ and $C = \dfrac{RR_C(X)}{RR_U(X)}$ is constant for $X > 0$.

Then:

$$B = \frac{PAF_L}{PAF}$$

$$= \frac{E_X(RR_C(X)) - 1}{E_X(RR_C(X))} \frac{E_X(RR_U(X))}{E_X(RR_U(X)\frac{RR_C(X) - 1}{RR_C(X)})}$$ (equations (7), (8), main manuscript)

$$= \frac{1 - \pi + \pi E_{X|X>0}(RR_C(X)) - 1}{1 - \pi + \pi E_{X|X>0}(RR_C(X))} \frac{1 - \pi + \pi E_{X|X>0}(RR_U(X))}{\pi E_{X|X>0}(RR_U(X)\frac{RR_C(X) - 1}{RR_C(X)})}$$

$$= C \frac{1 - \pi + \pi E_{X|X>0}(RR_U(X))}{1 - \pi + \pi E_{X|X>0}(RR_C(X))}$$

$$= C \frac{1 + \pi(E_{X|X>0}(RR_U(X)) - 1)}{1 + \pi(C \times E_{X|X>0}(RR_U(X)) - 1)}$$

where the second last and last lines follow from the assumption that $RR_C(X) = C \times RR_U(X)$ when $X > 0$. The proof of equation (2) is almost identical.

S10. Expressing, $RR_C = \dfrac{P(Y_1 = 1)}{P(Y_0 = 1)}$**,** $RR_e = \dfrac{P(Y_1 = 1 \mid X = 1)}{P(Y_0 = 1 \mid X = 1)}$ **as weighted averages of** $RR_C(c)$**.**

Here we again assume that conditional exchangeability: $Y_x \perp X \mid C = c$, holds within each confounder strata c, for $x \in \{0,1\}$, and consistency: If $X = x$, $Y = Y_x$, for $x \in \{0,1\}$

$$RR_C = \frac{P(Y_1 = 1)}{P(Y_0 = 1)}$$

$$= \frac{E_C\{P(Y_1 = 1 \mid C)\}}{E_C\{P(Y_0 = 1 \mid C)\}}$$

$$= \frac{E_C\{P(Y_1 = 1 \mid X = 1, C)\}}{E_C\{P(Y_0 = 1 \mid X = 0, C)\}} \text{ (By exchangeability)}$$

$$= \frac{E_C\{P(Y = 1 \mid X = 0, C)\}}{E_C\{P(Y = 1 \mid X = 1, C)\}} \text{ (By consistency)}$$

$$= \frac{E_C\{(P(Y = 1 \mid X = 0, C) RR_C(C))\}}{E_C\{P(Y = 1 \mid X = 0, C)\}}$$

Similarly, we can show that

$$RR_e = \frac{E_{C|X=1}\{P(Y = 1 \mid X = 0, C) RR_C(C)\}}{E_{C|X=1}\{P(Y = 1 \mid X = 0, C)\}}$$

Relative and absolute bias of the unadjusted Levin formula

$$PAF = [\frac{\pi RR_U}{1+\pi(RR_U-1)}]\frac{RR_C-1}{RR_C}$$

$$PAF_{L,U} = \frac{\pi(RR_U-1)}{1+\pi(RR_U-1)}$$

$$\implies \frac{PAF_{L,U}}{PAF} = \frac{RR_C}{RR_C-1}\frac{RR_U-1}{RR_U} = C\frac{RR_U-1}{CRR_U-1}$$

Expressing RR_U in terms of RR_C

$$RR_U = \frac{P(Y=1\,|\,X=1)}{P(Y=1\,|\,X=0)}$$

$$= \frac{E_{C|X=1}P(Y=1\,|\,X=1,C)}{E_{C|X=0}P(Y=1\,|\,X=0,C)}$$

$$= \frac{E_{C|X=1}(RR(C)P(Y=1\,|\,X=0,C))}{E_{C|X=0}P(Y=1\,|\,X=0,C)}$$

Under the assumption of no effect modification, this simplifies to $RR(c) = RR_C$ for all c

$$= RR_C \times \frac{E_{C|X=1}P(Y=1\,|\,X=0,C)}{E_{C|X=0}P(Y=1\,|\,X=0,C)}$$
